# Comparative genomics of hospital-associated vancomycin-resistant *Enterococcus faecium* from regional tertiary hospitals in Thailand

**DOI:** 10.7717/peerj.21354

**Published:** 2026-06-03

**Authors:** Chotiwit Sittiyuno, Mingkwan Yingkajorn, Sarunyou Chusri, Komwit Surachat, Arnon Chukamnerd, Amornrat Sianglum, Jiraphorn Nilsakul, Pimonsri Mittraparp-arthorn, Wipawadee Sianglum

**Affiliations:** 1 Division of Biological Science, Faculty of Science, Prince of Songkla University, Hat Yai, Songkhla, Thailand; 2 Department of Pathology, Faculty of Medicine, Prince of Songkla University, Hat Yai, Songkhla, Thailand; 3 Division of Infectious Diseases, Department of Internal Medicine, Faculty of Medicine, Prince of Songkla University, Hat Yai, Songkhla, Thailand; 4 Department of Biomedical Sciences and Biomedical Engineering, Faculty of Medicine, Prince of Songkla University, Hat Yai, Songkhla, Thailand; 5 Translational Medicine Research Center, Faculty of Medicine, Prince of Songkla University, Hat Yai, Songkhla, Thailand; 6 Clinical Microbiology Laboratory, Department of Medical Technology and Clinical Pathology, Sunpasitthiprasong Hospital, Mueang, Ubon Ratchathani, Thailand

**Keywords:** Antibiotic resistance, Vancomycin-resistant *Enterococcus faecium*, Virulence factors, Whole-genome sequencing, Multidrug resistance

## Abstract

**Background:**

Vancomycin-resistant *Enterococcus faecium* (VREfm) poses a significant public health threat due to increasing antimicrobial resistance and virulence factors associated with severe infections. The primary objective was to investigate the genetic and phenotypic characteristics of 46 VREfm clinical isolates obtained from three tertiary hospitals in Thailand (2015-2019).

**Methods:**

Phenotypic testing included antimicrobial susceptibility testing (AST) and virulence factor testing (gelatinase, lipase, protease, hemolytic activity, and biofilm formation), which were performed using standard laboratory methods. Short-read whole-genome sequencing (WGS) was performed to determine sequence types (ST), identify antimicrobial resistance and virulence genes, and perform phylogenetic analysis using SNP alignments.

**Results:**

All isolates exhibited vancomycin and teicoplanin resistance, and γ-hemolysis was the predominant pattern (23 isolates). Biofilm formation was predominantly moderate to weak. Most isolates had multiple antimicrobial resistance and virulence-associated genes. Genotype and phenotype concordance were high for β-lactams, ciprofloxacin, and glycopeptides, whereas tetracycline (54.35%), streptomycin (45.65%), and gentamicin (45.65%) exhibited moderate concordance. Phylogenetic analysis revealed two major clades, Clade I (predominantly ST80) and Clade II (predominantly ST17). One isolate represented a novel ST, ST2331 (Clade I), which was identified from urine.

**Conclusion:**

VREfm isolates from the three hospitals showed multidrug-resistant profiles and carried various resistance and virulence-associated genes. Whole-genome sequencing revealed two major phylogenetic clades. Overall, genotypic findings corresponded well with the phenotypic resistance patterns, with a small number of isolates showing genotype-phenotype discrepancies. These results highlight the genomic diversity and clinical relevance of VREfm circulating in Thai tertiary hospitals.

## Introduction

Antimicrobial resistance (AMR) has become one of the most critical global public health threats. Prolonged use and overuse of antimicrobial agents in healthcare and the community contributed to the emergence of multidrug-resistant (MDR) pathogens and reduced the effect of antimicrobial agents (Cornejo-Juárez et al., 2015; Wang et al., 2019). MDR organisms cause higher mortality and morbidity. The World Health Organization (WHO) highlights antimicrobial stewardship, surveillance to contain resistance, and preserving the therapeutic effect (Fahim, 2021). Gram-positive bacteria, including methicillin-resistant *Staphylococcus aureus* (MRSA) and vancomycin-resistant enterococci (VRE), are major MDR challenges in hospital and community settings (Siegel et al., 2022). MDR in *Enterococcus* species is typically defined as resistance to at least one agent in three or more antimicrobial classes, which significantly limits therapeutic options (Magiorakos et al., 2012).

*Enterococcus* spp. are facultatively anaerobic, Gram-positive cocci that colonize the gastrointestinal tract of humans and animals and persist in environments (Gholizadeh et al., 2020; Pillay, Zishiri & Adeleke, 2018; Sianglum et al., 2009). *Enterococcus faecium* and *Enterococcus faecalis* are important causes of opportunistic and nosocomial infections, including urinary tract infections, bacteremia, endocarditis, and wound infections (Biggel et al., 2021; Fiore, Van Tyne & Gilmore, 2019). *E. faecium* and *E. faecalis* possess a combination of intrinsic and acquired antimicrobial resistance mechanisms that contribute to their being hospital-associated pathogens (Arias & Murray, 2012). Several epidemiological factors cause VRE transmission in healthcare facilities, including prolonged hospitalization, receiving high doses of antibiotics, immunocompromised hosts, close contact with the patient, and contaminated environments (Biggel et al., 2021; Iseppi et al., 2020; Knudsen et al., 2022).

VRE are common hospital-acquired pathogens, accounting for the prevalence of VRE in Europe (19%) (Biggel et al., 2021), North America (21%) (Bezdicek et al., 2023), and Asia (8–24%) (Shrestha et al., 2021). In Thailand, the prevalence of vancomycin-resistant *Enterococcus faecium* (VREfm) increased from 3.2–12.8%, and VREfs increased from 0.1–1.5% in 2013–2022 (National Antimicrobial Resistance Surveillance Center T, 2022). The prevalence of VRE infections in hospitals may be attributed to their ability to tolerate high temperatures, chlorine, and alcohol solutions (Arias & Murray, 2012). Enterococcal infections, including urinary tract infections, bacteremia, intra-abdominal infections, and endocarditis, are frequently diagnosed in hospitalized patients (Fiore, Van Tyne & Gilmore, 2019). WHO has classified VREfm as a high-priority pathogen requiring immediate therapeutic research and development due to its significant impact on clinical and public health (Ayobami et al., 2020). *Enterococcus faecium* exhibits resistance to multiple antibiotics, including β-lactams, aminoglycosides, and cephalosporins, through low-affinity penicillin-binding proteins and efflux mechanisms (Bezdicek et al., 2023). The mobile genetic elements, such as plasmids and transposons, not only support antimicrobial resistance but also allow efficient horizontal gene transfer to other bacteria (Arias & Murray, 2012; Aun et al., 2021). The acquisition of resistance to glycopeptides (notably vancomycin and teicoplanin) poses a significant challenge for the treatment of enterococcal infections. Nine glycopeptide-resistance operons (vanA, vanB, vanC, vanD, vanE, vanG, vanL, vanM, and vanN) have been identified phenotypically and genotypically. *vanA* and *vanB* are the most clinically relevant, conferring high-level resistance through modification of the D-Ala–D-Ala terminus to D-Ala–D-Lac (Bender et al., 2018). The *vanA*, *vanR*, *vanS*, *vanH*, *vanA*, *vanX*, *vanY,* and *vanZ* genes are typically located on the transposon Tn1546, promoting dissemination among strains (Saengsuwan et al., 2021). *E. faecium* isolates frequently carry *vanA*, whereas *E. faecalis* contains *vanA* or *vanB*, depending on local epidemiology (Saengsuwan et al., 2021).

The increase in enterococci as hospital-acquired pathogens was caused by their ability to survive antimicrobial agents. Bacterial virulence factors contribute to pathogenesis, antimicrobial resistance, survival in the environment, colonization, and dissemination (Tendolkar et al., 2004). Enterococci possess many genes responsible for producing adhesion proteins that promote attachment to host tissues. Key virulence factors include cytolysin (*cyl*), a hemolytic toxin (Semedo et al., 2003), and gelatinase (*gelE*), an extracellular protease involved in tissue degradation (Hamid Kazemian et al., 2019). Furthermore, surface proteins such as hyaluronidase (*hyl*) and enterococcal surface protein (*esp*) play crucial roles in adhesion and biofilm formation (Golinska et al., 2013; Hashem, Abdelrahman & Aziz, 2021). Other contributing factors include *elrA*, *ebpA*, and sex pheromone plasmids (*cob, cpd,* and *ccf*), which collectively enhance bacterial persistence and virulence (Nunez et al., 2018; Kim, Rosa & Min, 2020; Pillay, Zishiri & Adeleke, 2018). Their ability to form biofilms is an essential pathogenic characteristic. This characteristic enhances surface colonization and facilitates adherence to host cells. Biofilm production, supported by extracellular polymeric substances (EPS), protects bacteria from host immune responses, and antimicrobial agents and facilitates horizontal gene transfer (Khalil et al., 2023; Melese, Genet & Andualem, 2020).

In the past decade, whole-genome sequencing (WGS) has revolutionized enterococcal epidemiology. WGS provides high-resolution data to characterize genetic diversity, resistance and virulence determinants, and clonal relationships (Brodrick et al., 2016; Eichel et al., 2020; Nijhuis et al., 2021). Integration of genomic and phenotypic data allows assessment of genotype-phenotype concordance, elucidating underlying mechanisms of resistance and virulence (Fujikura et al., 2019; Raven et al., 2016). Due to VRE prevalence in Thailand and limited genomic data from regional hospitals, a comprehensive study integrating phenotypic and genomic analysis is essential. The objective of our study was to analyze the phenotype characteristics and genomes of vancomycin-resistant enterococci in healthcare settings from three tertiary hospitals in Northeast and Southern Thailand, using genotype-phenotype correlations to inform infection control and stewardship strategies.

## Materials & Methods

### Bacterial isolate collection

All 46 isolates obtained were identified as vancomycin-resistant *Enterococcus faecium* (VREfm). Enterococci isolates were leftover samples previously isolated from various clinical specimens, including urine, tissue, pus, body fluids, sputum, rectal swabs, and hemocultures, at the Clinical Microbiology Laboratory of three hospitals. Twenty-five isolates were collected from super tertiary care at Songklanagarind Hospital (SKA-PSU) in Songkhla, Southern Thailand, from 2018 to 2019. Twelve isolates were obtained from tertiary care at Hat Yai Hospital (HTY) in Songkhla, Southern Thailand, and nine isolates were isolated from Sunpasitthipasong Hospital (SPS), the main hospital, classified under the Ministry of Public Health as a regional hospital of Ubon Ratchathani, Northeastern Thailand. VREfm isolates from both hospitals were obtained during 2015–2018.

### Antimicrobial susceptibility test

Antimicrobial susceptibility testing of the VREfm isolates was performed using a disk diffusion test and broth microdilution, according to the Clinical and Laboratory Standards Institute (CLSI) guidelines (Clinical, and Institute LS, 2020). The following antimicrobial disks were included (Oxoid, Basingstoke, UK): ampicillin (AP), ciprofloxacin (CIP), chloramphenicol (C), gentamycin (CN), linezolid (LZD), penicillin (PG), streptomycin (S), teicoplanin (TEC), tetracycline (TE), and vancomycin (VA). The broth microdilution method was used to evaluate the minimal inhibitory concentration (MIC) of vancomycin, levofloxacin, and fosfomycin. Briefly, overnight cultures were adjusted to a 0.5 McFarland standard and diluted 1:100 in Mueller-Hinton broth (MHB). Inoculum was transferred to 96-well plates containing serial two-fold dilutions of vancomycin (2–1,024 µg/ml), levofloxacin (0.5–256 µg/ml), and fosfomycin (32–1,024 µg/ml) in MHB. The MIC value was defined by the observation of the concentration that did not show any bacterial growth, in contrast to the control. *E. faecalis* ATCC29212 and *E. faecium* DMST14756 were used for positive control strains.

### Determination of virulence factors

#### Hemolytic activity

Hemolytic activity of all isolates was evaluated using a modified method based on that of Semedo et al. (2003). Bacterial isolates were inoculated on blood agar at 35 ± 2 °C overnight and subsequently assessed for hemolysis patterns. β-hemolysis by a clear zone, α-hemolysis was identified by a green zone surrounding the colonies, and γ-hemolysis by the absence of hemolytic activity. *Staphylococcus aureus* ATCC25923 was used as a positive control strain for β-hemolysis.

#### Lipase activity

Lipase assay was performed using a modified method based on that of Biswas et al. (2014), using egg yolk agar. VREfm isolates were streaked onto the medium and incubated at 35 ± 2 °C for 24–48 h. The appearance of an opaque zone around colonies showed the positive test result. *S. aureus* ATCC25923 was used as a positive control strain.

#### Protease activity

A protease assay was conducted using skim milk agar (Biswas et al., 2016). After inoculating the media with VREfm isolates, plates were incubated at 35 ± 2 °C for 24 h. A transparent zone surrounding the colonies provided evidence of protease activity. *S. aureus* ATCC25923 was used as a positive control strain.

#### Gelatinase activity

Gelatinase activity of the isolates was evaluated using nutrient gelatin agar (HiMedia, Mumbai). After that, the plate was incubated at 35 ± 2 °C for 24 h and refrigerated at 4 °C for 30 min. Liquefaction of gelatin was considered a positive, according to Biswas et al. (2016) method (Fahmy et al., 2021). *S. aureus* ATCC25923 was used as a positive control strain.

### Biofilm formation

Biofilm formation was evaluated using a crystal violet assay as described previously (Rosdee et al., 2025; Naclerio, Onyedibe & Sintim, 2020; Singh et al., 2017). The VREfm isolates were inoculated in a brain heart infusion broth containing 0.25% glucose (GBHI) and incubated at 35 ± 2 °C for 24 h. The overnight culture was diluted with 0.85% NaCl until the turbidity reached 0.5 MacFarland (10^8^ CFU/ml). The suspensions were inoculated with a final volume of 200 µl and incubated at 35 ± 2 °C for 24 h. 96-well plates were washed twice using 200 µl phosphate-buffered saline (PBS) and air-dried for 15 min. The remaining adherent microorganisms were stained with 0.1% crystal violet solution for 20 min, and the solution was then discharged. The plates were dried at 35 ± 2 °C for 10 min and washed twice using 200 µl PBS. After air-drying, dye solubilization was performed by adding 200 µl of acetone: ethanol mixture (20:80, v/v) in each well. The optical density (OD) measurement used with a microplate reader (Enspire™, Perkin Elmer, Waltham, MA, USA) at a wavelength of 570 nm. *S. aureus* ATCC25923 was used as a positive control strain. The results were generated using the measurement of optical density to interpret biofilm formation. Strains were classified as follows: OD_sample_ > 4OD_negative_ was interpreted as a strong biofilm former, 4 OD_negative_ ≥ OD_sample_ > 2 OD_negative_ were interpreted as a moderate biofilm former, 2 OD_negative_ ≥ OD_sample_ > OD_negative_ were interpreted as a weak biofilm former, and OD_sample_ < OD_negative_ were interpreted as a non-biofilm former (Khwankaew et al., 2026; Naclerio, Onyedibe & Sintim, 2020).

### The genome analysis

Bacterial genomic DNA was extracted using the OMEGA BIOTEK DNA extraction kit (Bangkok Genomics Innovation Co., Ltd.). The Bacterial DNA sample was performed with short-read whole-genome sequencing (WGS) was conducted on the MGISEQ-2000 platform with 150-bp paired-end reads (Zhong et al., 2019). The Galaxy Australia platform (https://usegalaxy.org.au/) was utilized for data analysis, and raw paired-end sequencing reads were assessed for quality control using FastQC. The validated reads were trimmed with Trimmomatic to remove adapters and low-quality bases and de novo assembled using SPAdes (Brodrick et al., 2016; Leigh et al., 2022). The assemblies were annotated, and gene sequences were predicted using Prokka (Rubio, Jimenez & Perez-Pulido, 2022). Antimicrobial resistance genes were identified with ResFinder (4.1) using the *Enterococcus faecium* database from the Center for Genomic Epidemiology (CGE: https://www.genomicepidemiology.org/) with the Comprehensive Antibiotic Resistance Database (CARD: https://card.mcmaster.ca/). The presence of virulence factors was assessed with Virulence Finder (software version 2.0.3) from CGE and the Virulence Factor Database (VFDB: http://www.mgc.ac.cn/VFs/main.htm). Acquired antimicrobial resistance genes, chromosomal resistance-associated mutations, and virulence-associated genes were identified using minimum thresholds of ≥90% sequence identity and ≥60% sequence coverage. Integrons were discovered using IntegronFinder on Galaxy Australia, and sequence types (STs) using MLST allele sequence from MLST (software version 2.0.9) from CGE. CRISPR-CasFinder and PHASTER were used to identify Clustered Regularly Interspaced Short Palindromic Repeats (CRISPR) associated with Cas loci and bacteriophage. Single-nucleotide polymorphisms (SNPs) were identified using Snippy (Galaxy Version 4.6.0) on Galaxy Australia, with a minimum sequencing depth of 10 × and at least 90% coverage of the reference genome. All *E. faecium* isolates were mapped against the *E. faecium* reference genome GCF_009697285.1 (ASM969728v1), downloaded from the National Center for Biotechnology Information (NCBI) Assembly database. The snippy-core tool (Galaxy Version 4.1.0) merged Snippy outputs across all isolates to produce a core SNP alignment and a core full alignment. The resulting core.full.aln file was then processed with snippy-clean_full_aln to a cleaned alignment. The recombination-filtered alignment was produced using Gubbins and was then used as input for IQ-TREE (Galaxy Version 2.2.2). Phylogenetic trees generated by IQ-TREE were visualized and annotated using the Interactive Tree of Life (iTOL), with the final tree being midpoint rooted. Proksee tool (https://proksee.ca/) was used to calculate the average nucleotide identity (ANI) number of the isolates to measure nucleotide-level genomic similarity between the coding regions of two genomes. Pan-genome analysis was visualized by Phandango software (version 1.3.1) (Dubin et al., 2019; Hadfield et al., 2018; Knudsen et al., 2022; Neumann et al., 2020).

### Statistical analysis

The association between biofilm formation and the *empB*, *empC*, *esp*, and *hyl* genes was analyzed using the Chi-square test. Statistical analysis was performed by SPSS software, and *p* ≤ 0.05 was considered statistically significant (Biswas et al., 2016; Hashem, Abdelrahman & Aziz, 2021).

### Data availability statement

The assembled genomes of all VREfm isolates have been deposited in the NCBI GenBank under BioProject number PRJNA1301073 with BioSample numbers SAMN50434509 to SAMN50434554.

### Ethical statement

The present work has been reviewed by the Human Research Ethics Committee, Faculty of Medicine, Prince of Songkla University (REC. 64-457-19-2), which determined that the research met the criteria for exemption determination, and the Institutional Review Board waived informed consent.

## Results

### Bacterial population phylogenetic analysis

**Figure 1:**
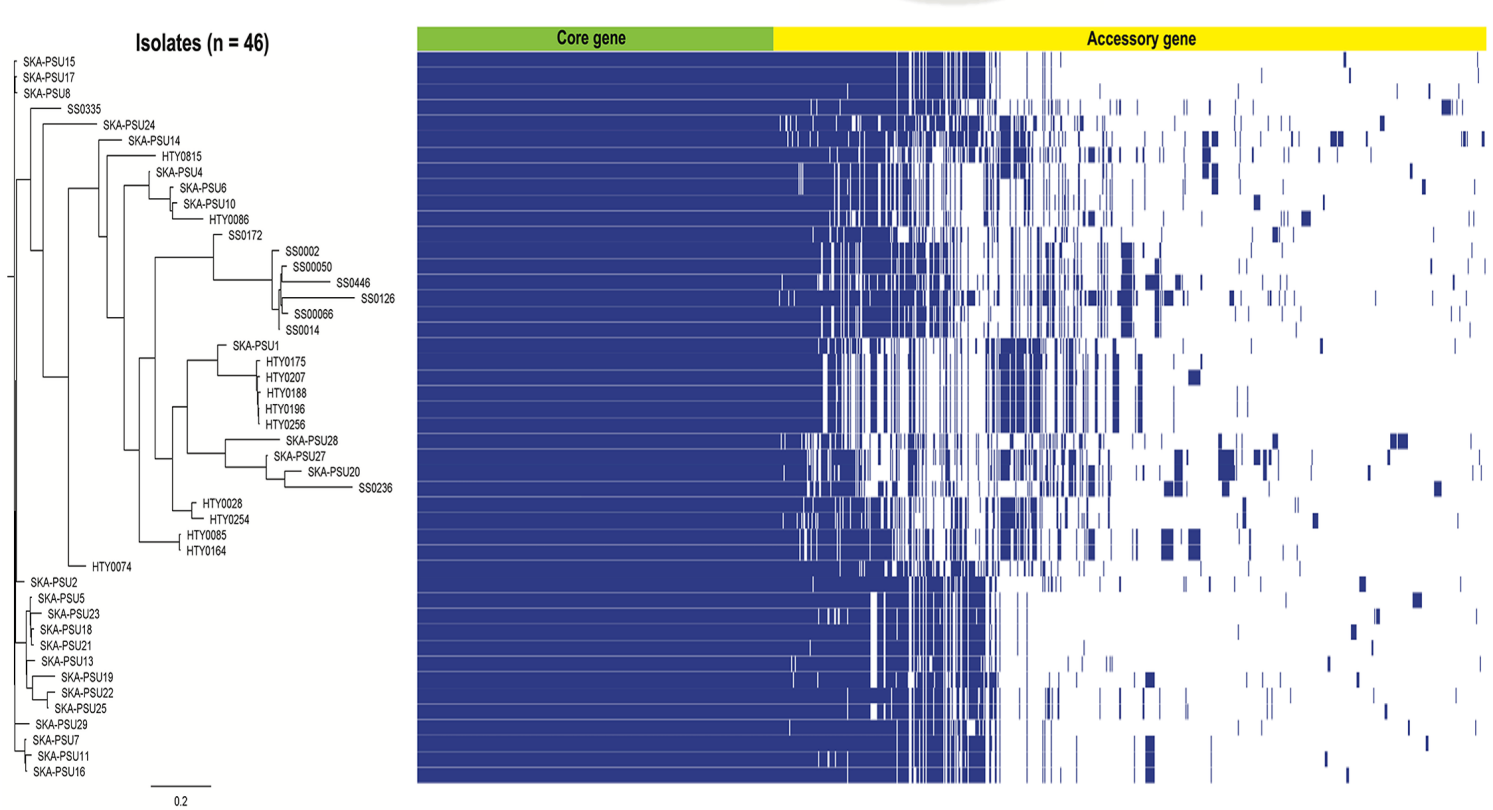
Genetic relatedness analysis of vancomycin-resistant *Enterococcus faecium*. The accessory-based phylogenetic tree and pie chart against the pan-genes. The core and accessory genes were distributed in the phylogenetic tree. The blue color indicates the presence of genes, and the pan-genome was visualized using Roary.

**Figure 2:**
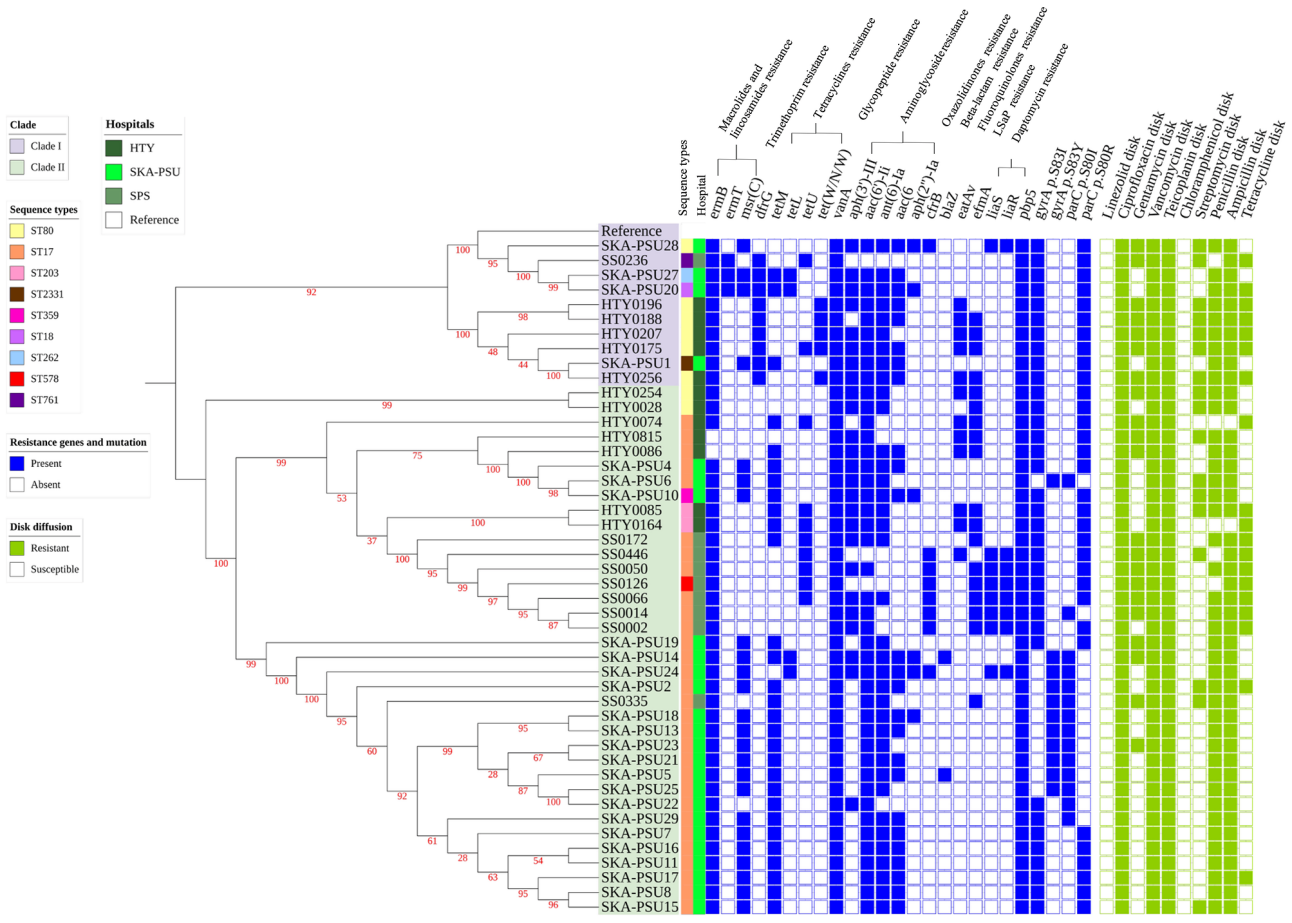
Maximum-likelihood phylogeny of 46 *Enterococcus faecium* isolates with corresponding sequence types, antimicrobial resistance genes, resistance-associated mutations, and phenotypic antimicrobial susceptibility profiles. A maximum-likelihood phylogenetic tree was constructed using a recombination-filtered core SNP alignment generated through Snippy, Snippy-core, and Gubbins, with *E. faecium* reference genome GCF_009697285.1. Tree inference was performed using IQ-TREE with ultrafast bootstrap support values shown at internal nodes. The phylogenetic tree was visualized and annotated using iTOL. Two major phylogenetic groups, designated as Clade I and Clade II, were observed. Antimicrobial resistance genes and mutations were identified using thresholds of ≥ 90% sequence identity and ≥ 60% coverage. The heatmap alongside the tree displays the sequence types (STs), presence/absence of antimicrobial resistance genes, and corresponding phenotypic disk diffusion results for each isolate.

**Figure 3:**
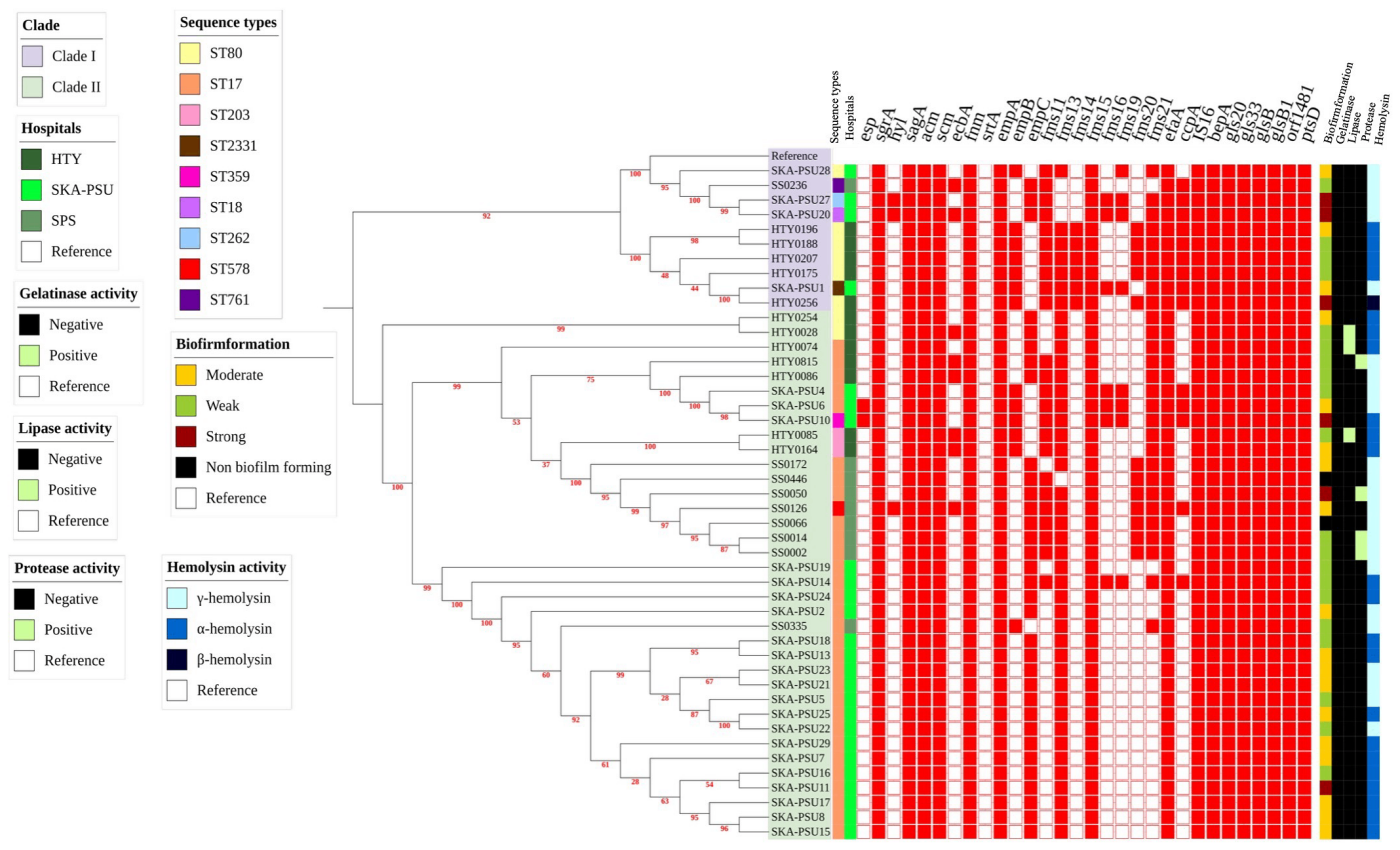
Maximum-likelihood phylogeny of 46 *Enterococcus faecium* isolates with corresponding virulence gene profiles and phenotypic virulence characteristics. A maximum-likelihood phylogenetic tree was reconstructed using a recombination-filtered core SNP alignment generated from Snippy, Snippy-core, and Gubbins. Tree inference was performed with IQ-TREE, and bootstrap support values are shown on internal branches. The *E. faecium* reference genome (GCF_009697285.1) was used as a reference. The phylogenetic tree was visualized and annotated using iTOL. Two major phylogenetic groups, Clade I and Clade II, were identified. Virulence-associated genes were identified using thresholds of ≥ 90% sequence identity and ≥ 60% coverage. The accompanying heatmap displays the distribution of virulence-associated genes and the corresponding phenotypic virulence factors for each isolate.

VREfm isolates were previously collected from clinical specimens, including urine (*n* = 34, 73.91%), tissue (*n* = 2, 4.35%), pus (*n* = 4, 8.70%), body fluid (*n* = 2, 4.35%), sputum (*n* = 1, 2.17%), rectal swab (*n* = 2, 4.35%), and hemoculture (*n* = 1, 2.17%). The accessory-based phylogenetic tree of pan-genome profiles among the studied isolates revealed that, among 5,564 pan genes, 1,882 (33.82%) and 3,682 (66.18%) genes were classified as core and accessory genes, respectively. Moreover, we found that in 2,954 genes encoding for hypothetical proteins, 547 (18.52%) and 2407 (81.48%) genes were observed in core and accessory genes, respectively (Fig. 1). The ANI values ranged between 98.99% and 100%, which shows all VRE isolates were intra-species of VREfm. Multilocus sequence typing identified nine sequence types (STs) among the isolates (Table 1, Table S1). ST17 was the predominant lineage in SKA-PSU Hospital (20/25; 80%) and SPS Hospital (7/9; 78%), whereas ST80 was the most common ST in HTY Hospital (7/12; 58%). A novel sequence type, ST2331, was identified in SKA-PSU and submitted to PubMLST; allelic comparison showed that this lineage is closely related to ST80 (Table S10). A maximum-likelihood phylogenetic tree based on core-genome SNPs separated the isolates into two major clades (Figs. 2 and 3; Fig. S1). Clade I included isolates belonging to ST80, ST18, ST262, ST761, and the novel ST2331, whereas Clade II comprised all ST17 isolates and 4 isolates from other STs, namely, SS0126 (ST578), SKA-PSU10 (ST359), HTY0085 (ST203), and HTY0164 (ST203), which also clustered within this clade (Tables S10 and S1). Clinical specimens were distributed throughout both clades without clustering, indicating that SNP-defined lineages are not associated with specific infection sites and can cause infection across multiple specimen types.

**Table 1:** Antimicrobial profile, virulence factors, and multi-locus sequence typing (MLST) of VREfm isolates from three tertiary hospitals. Songklanagarind Hospital (SKA-PSU), Hat Yai Hospital (HTY), and Sunpasitthipasong Hospital (SPS).

Disk	Hospitals (%)		
	SKA-PSU (*n* = 25)	SPS (*n* = 9)	HTY (*n* = 12)	
Antimicrobial susceptibility test			
Vancomycin	100	100	100	
Teicoplanin	100	100	100	
Ampicillin	100	100	83.3	
Penicillin	100	66.7	83.3	
Ciprofloxacin	100	100	100	
Gentamycin	16	88.9	75	
Streptomycin	20	33.3	75	
Tetracycline	12	88.9	66.7	
Chloramphenicol	0	0	0	
Linezolid	0	0	0	
Virulence factors test			
Weak-biofilm producer	36	44.4	25	
Moderate-biofilm producer	52	22.2	25	
Strong-biofilm producer	12	11.1	8.3	
γ-hemolytic	48	100	16.7	
α-hemolytic	52	0	75	
β-hemolytic	0	0	8.3	
Lipase producer	0	0	25	
Protease producer	0	33.3	8.3	
Gelatinase producer	0	0	0	
Sequence type (ST)			
ST17	80.0	77.8	25.0	
ST80	4.0	0.0	58.3	
ST203	0.0	0.0	16.7	
ST18	4.0	0.0	0.0	
ST262	4.0	0.0	0.0	
ST359	4.0	0.0	0.0	
ST578	0.0	11.1	0.0	
ST761	0.0	11.1	0.0	
ST2331	4.0	0.0	0.0	

### Antimicrobial resistance profiles and genotypes across phylogenetic clades

Phenotypic and genotypic analysis revealed that antimicrobial resistance patterns were conserved mainly across the two major phylogenetic clades, with specific variations driven by hospital origin. Resistance to glycopeptides and fluoroquinolones was observed in all VREfm isolates from the three hospitals. Specifically, all isolates from SKA-PSU (25/25), HTY (12/12), and SPS (9/9) were resistant to vancomycin, teicoplanin, and ciprofloxacin (100%). This phenotype was genetically supported by the presence of the *vanA* gene in all isolates and consistent mutations in the quinolone resistance-determining regions (QRDR). Most isolates, particularly those in Clade I (ST18, ST80, ST262, ST761, and ST2331), harbored the characteristic gyrA S83I and parC S80R substitutions, while a small number of Clade II isolates presented gyrA S83Y and parC S80I (Fig. 2, Table S6).

β-lactam resistance was also widespread, with resistance to ampicillin in SKA-PSU and SPS isolates (100%), lower rates in HTY (83.3%), and resistance to penicillin in SKA-PSU isolates (100%) and lower rates in HTY (83.3%) and SPS (66.7%). In contrast, gentamicin and streptomycin resistance varied across hospitals. SPS showed high resistance rates (88.9% and 33.3%), followed by HTY (75% and 75%), whereas SKA-PSU exhibited the lowest levels (16% and 20%). Tetracycline resistance similarly differed, with SPS and HTY showing high resistance (88.9% and 66.7%), while SKA-PSU demonstrated markedly lower resistance (12%). All isolates across the three hospitals remained susceptible to chloramphenicol and linezolid. Genotypically, *vanA* was present in isolates from all hospitals, correlating with the glycopeptide-resistant phenotype. Tetracycline-resistance genes (*tetM*, *tetU*) were common in HTY and SPS, whereas SKA-PSU harbored only *tetM*, consistent with the lower tetracycline resistance phenotype. Aminoglycoside-resistance determinants were widely distributed but varied in composition. SPS and HTY carried multiple aminoglycoside-modifying enzyme (AME) genes, *aph(3′)-III*, *aac(6′)-Ii*, *ant(6)-Ia*, and *aac(6′)-aph(2″)*, supporting their high-level aminoglycoside resistance patterns, whereas SKA-PSU isolates, although genetically equipped with several AME genes, displayed limited phenotypic resistance. For macrolides, resistance determinants were ubiquitous; *ermB* and *ermT* were present in isolates from all hospitals, and *msrC* was consistent with the profiles typical of *E. faecium* (Fig. 2, Tables S2 and S4).

### Determination of the virulence factor

All VREfm isolates demonstrated the ability to form biofilms, though the distribution of biofilm strength varied by region. SKA-PSU isolates showed the highest proportion of moderate biofilm producers (52%), whereas HTY isolates predominantly exhibited weak biofilm formation (66.7%). SPS isolates presented an intermediate pattern, with weak biofilm in 44.4% and moderate biofilm in 22.2% of isolates. Strong biofilm formation was detected in all hospitals, but at low frequencies (HTY 8.3%, SPS 11.1%, and SKA-PSU 16%). Hemolytic patterns varied among isolates from the three hospitals. HTY isolates showed α-hemolysis (75%) and β-hemolysis (8.3%). SPS isolates showed γ-hemolysis, whereas SKA-PSU isolates showed α-hemolysis (52%). Protease and lipase activities were not observed. HTY isolates expressed low levels of protease (8.3%) and lipase (25%), and SPS isolates showed protease in 33.3% of isolates. Genotypically, all three hospitals were observed to have virulence genes together, including *efaA*, *acm*, *scm*, *sgrA*, *sagA*, *empABC*, *ecbA*, and *ccpA*, and broad sets of adhesion-related genes, including *fms* and *gls*. These genes are associated with adherence, colonization, immune evasion, and biofilm formation, and were consistently found across all isolates (Fig. 3, Tables S3 and S5).

### CRISPR-Cas loci, integrons, and prophage analysis

**Figure 4:**
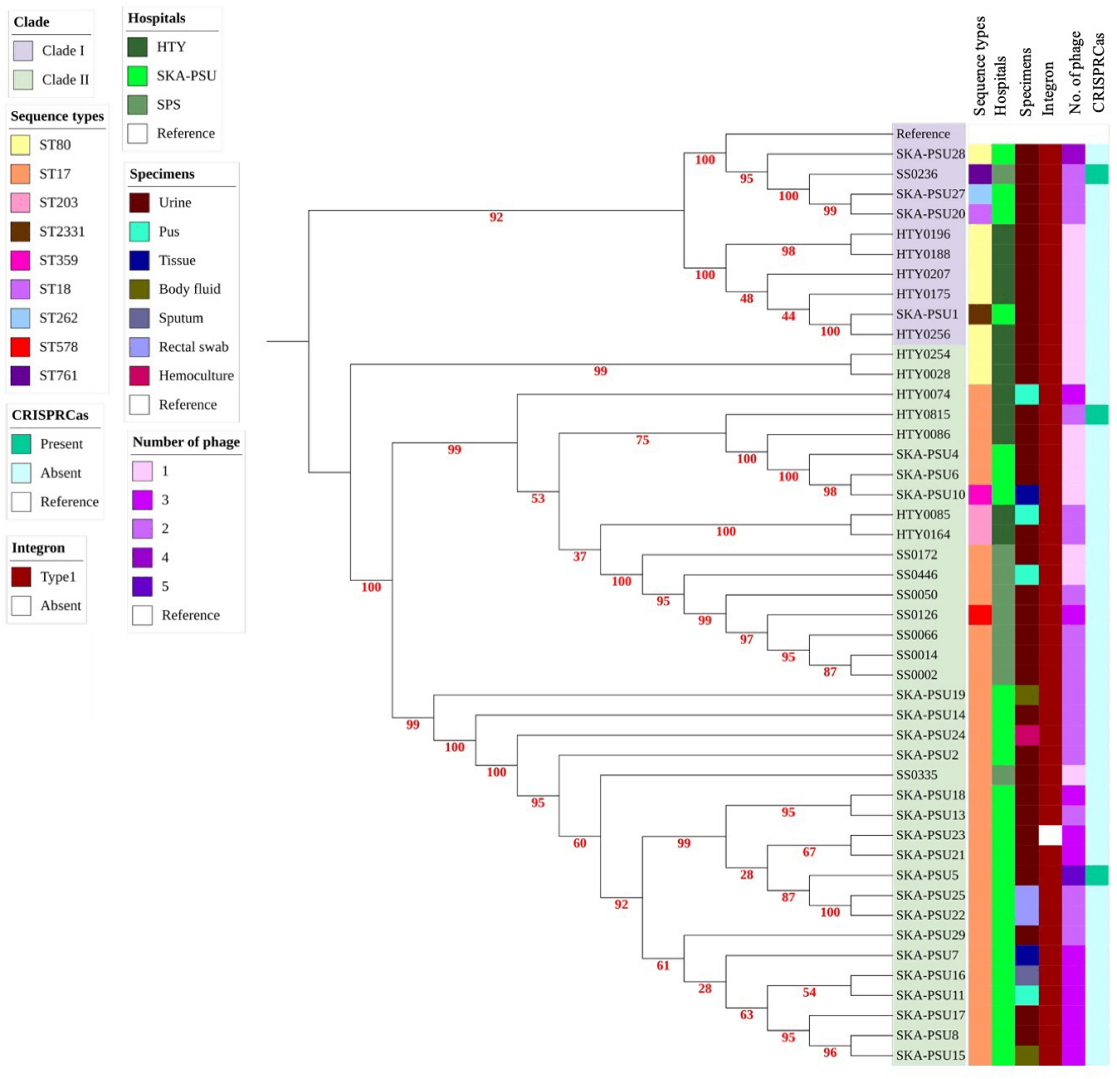
Maximum-likelihood phylogenomics of 46 *Enterococcus faecium* isolates with associated metadata, including sequence types, hospital sources, clinical specimen types, phage, integrons, and CRISPR-Cas. A maximum-likelihood phylogenetic tree was reconstructed using a recombination-filtered core SNP alignment generated by Snippy, Snippy-core, and Gubbins. Tree inference was performed using IQ-TREE, and bootstrap support values are displayed on internal branches. The tree was rooted using the reference genome (*E. faecium* GCF_009697285.1) and identified two major phylogenetic groups, Clade I and Clade II. The heatmap alongside the tree presents hospital sources, clinical specimen types, phage, integrons, and CRISPR-Cas for each isolate.

This study assessed the Clustered Regularly Interspaced Short Palindromic Repeats (CRISPR) associated with Cas loci, which contained the prophage. CRISPR-Cas and prophage of 46 *E*. *faecium* isolates were identified using CRISPR-CasFinder and PHASTER. Ten isolates showed identifiable CRISPR sequences, but three isolates had Class 1-Cas protein associated with their CRISPR loci, including SKA-PSU5, HTY815, and SS0236. Prophage analysis with PHASTER detected intact or questionable prophages, and all isolates contained prophages. Prophages varied in size from 14.6–55 Kb. The study found no correlation between CRISPR-Cas loci and prophages in the examined isolate genome. Most isolates had integrons type I except the SKA-PSU23 isolate, which did not detect any integrons (Fig. 4, Tables S7, S8, and S9).

## Discussion

We characterized vancomycin-resistant *Enterococcus faecium* (VREfm) isolates from three tertiary hospitals in Thailand, with most isolates obtained from urine, consistent with their role in healthcare-associated urinary tract infections and previous Thai and international reports (Melese, Genet & Andualem, 2020; Papadimitriou-Olivgeris et al., 2014; Saengsuwan et al., 2023; Saengsuwan et al., 2021). Whole-genome sequencing revealed substantial genomic diversity, including a high proportion of hypothetical protein-encoding genes and nine distinct sequence types, notably the emergence of a novel ST2331. The dominance of ST17 in SKA-PSU and SPS, and of ST80 in HTY, suggests hospital-specific or region-specific dissemination. Similar patterns have been described in Bangkok, where ST17 and ST203 were frequent epidemics (Wongnak et al., 2021), and in Europe, where ST17 and ST80 predominated in Germany and Switzerland (Abdelbary et al., 2019; Eichel et al., 2020; Xanthopoulou et al., 2020). Conversely, Correa-Martinez et al. (2020) reported a higher prevalence of ST203, highlighting geographic variability. Phylogenetic analysis was employed to stratify the isolates into two distinct populations. While Clade I (ST80) consistently displayed a multidrug-resistant phenotype typical of hospital-adapted lineages, Clade II (ST17) exhibited remarkable intra-clade plasticity, containing both highly resistant and susceptible sub-populations. This finding contrasts with the multidrug resistance classically defining high-risk clones (Willems et al., 2012; Top et al., 2008), but aligns with recent evidence of the high genomic plasticity inherent to *E. faecium* (Van Hal et al., 2021; Palmer et al., 2012). Together, the high ANI values and large accessory genome underscore the evolution, adaptability, and potential endemicity of specific VREfm clones in Thai healthcare settings.

SKA-PSU1, isolated from urine at Songklanagarind Hospital, belonged to the novel sequence type ST2331 and carried the *vanA* gene. The isolation of VREfm from urine, a site not part of the normal microbiota, suggests its role in a urinary tract infection (Agudelo Higuita & Huycke, 2016). The isolate had multiple resistance determinants, including aminoglycoside and macrolide resistance genes, consistent with its phenotypic multidrug resistance profiles. Virulence phenotyping indicated moderate biofilm formation and γ-hemolysis activity, while virulence gene profiling revealed the presence of several adhesion-associated loci and cell wall-associated loci. Genomic mobility elements were prominent, including one intact prophage, a type 1 integron, and two CRISPR loci lacking Cas genes, suggesting high genomic plasticity. In the phylogenetic tree, SKA-PSU1 is grouped within Clade I, forming a distinct cluster with other ST2331 isolates, supporting its emergence as a potentially evolving lineage within the Southern Thailand hospital environment.

Vancomycin-resistant *Enterococcus faecium* (VREfm) is widely recognized for its extensive antimicrobial resistance (Sanderson et al., 2020). Consistent with this study, all examined isolates displayed high-level resistance to vancomycin, teicoplanin, and ciprofloxacin, corresponding with the presence of the *vanA* gene and fluoroquinolone-associated mutations in *gyrA* and *parC*. All isolates met the criteria for multidrug resistance, consistent with reports from Iran showing *vanA* carriage among VRE isolates (Arshadi et al., 2018) and prior Thai studies indicating that *vanA*, but not *vanB*, is the dominant glycopeptide-resistance determinant in Thailand (Saengsuwan et al., 2023; Saengsuwan et al., 2021; Wongnak et al., 2021).

Aminoglycoside resistance was variable across the three hospitals. Gentamicin resistance was highest in SPS (88.9%) and HTY (75%) (predominantly associated with Clade II-ST17 and Clade I-ST80, respectively), consistent with reports from Egypt, Malaysia, and India showing similarly elevated rates (Arundathi et al., 2022; Diab et al., 2019; Moussa et al., 2019), whereas SKA-PSU exhibited a much lower prevalence (16%), in line with previous data from Southern Thailand (Arundathi et al., 2022). Streptomycin resistance was also most pronounced in HTY (75%), comparable to findings from Egypt and other global reports where 78–89% of isolates showed high-level resistance (Abd El Hamid et al., 2023; Diab et al., 2019). These phenotypes corresponded well with the presence of aminoglycoside-modifying enzymes, including *aph(3′)-III*, *aac(6′)-Ii*, *ant(6)-Ia*, and *aac(6′)-aph(2″)*, which are widely reported among clinical *E. faecium* (Chopjitt et al., 2023; Haghi, Lohrasbi & Zeighami, 2019;  Cetinkaya, Falk & Mayhall, 2000). Tetracycline resistance was also higher in SPS (88.9%) and HTY (66.7%) compared with SKA-PSU (12%), similar to global observations (Ahmadpoor et al., 2021), and supported by the detection of *tetM*, *tetU*, and *tetL*, representing both ribosomal protection and efflux mechanisms (Ayeni et al., 2016; Chopjitt et al., 2023; Huys et al., 2004). Macrolide resistance was dominated by *ermB* (86.3%), with *ermT* (5.9%), while efflux-mediated resistance, *msrC,* was common in SKA-PSU isolates (92%), in agreement with established erythromycin-resistance mechanisms (Ahmadpoor et al., 2021). Although linezolid-resistance genes (*cfrB*, *optrA*, *poxtA*) were detected in a small minority of isolates, no phenotypic resistance was observed, low resistance rates were reported in Thailand and Europe (Sun et al., 2020; Xanthopoulou et al., 2020). All isolates remained resistant to ciprofloxacin, consistent with global trends (Gorski et al., 2024)*,* and confirming multidrug-resistant (MDR) status as defined by resistance to ≥3 antimicrobial classes (Basak, Singh & Rajurkar, 2016; Fahmy et al., 2021). Together, these findings reveal substantial inter-hospital variation in specific resistance phenotypes while reaffirming the widespread MDR profile of VREfm circulating in Thailand.

In addition to increasing antimicrobial resistance, several virulence traits contribute to the pathogenicity of VREfm, including adhesion, colonization, toxin production, and biofilm formation (Shahi et al., 2020). A majority of the isolates were found to produce weak to moderate biofilm, and enzymatic activities (protease and lipase) were infrequent, while gelatinase was absent. Hemolytic patterns varied geographically. SPS isolates produced exclusively γ-hemolysis, whereas SKA-PSU and HTY isolates exhibited a mixture of α- and γ-hemolysis. Genomic analysis revealed a broad virulence gene repertoire across all isolates, including adhesins (*acm*, *scm*, *ecbA*, and *fnm*), LPxTG cell–surface proteins (*esp*, *sgrA*, and multiple *fms* genes), pili clusters (*ebpABC* and *srtAC*), and secreted factors such as *sagA*. IS16, a marker of hospital-adapted *E. faecium,* was universally detected, consistent with previous findings (Laukova et al., 2019; Werner et al., 2011; Zaghloul & El Halfawy, 2022). Although most virulence genes were widespread, *hyl* was confined to SKA-PSU (8%) and SPS (33.3%), and absent from HTY, whereas *esp* was detected only in SKA-PSU (12%). Biofilm production showed no significant association with *esp* (*p* = 0.942) but was significantly associated with *hyl* (*p* = 0.013). The *empA* gene was conserved across all isolates, whereas *empBC* distribution differed across hospitals but did not correlate with biofilm formation, consistent with earlier studies reporting no link between *empABC* and biofilm phenotype (Khattak et al., 2020). Collectively, these findings indicate that VREfm isolates from all three hospitals share a core set of virulence determinants typical of hospital-adapted lineages, with limited geographic variability in specific markers such as *hyl* and *esp.* The presence of virulence genes such as *sagA*, *sgrA*, and cytolysin genes (*cylA*, *cylB*, and *cylM*) was not detected in these isolates. Although phenotypic hemolysis was observed in our isolates, classical cytolysin (*cyl*) genes and other known extracellular degradative enzymes were absent from the WGS data, suggesting the involvement of alternative mechanisms. Specifically, α-hemolysis in *Enterococcus* is primarily driven by hydrogen peroxide (H_2_O_2_) accumulation rather than specific cytolysins (McDevitt et al., 2020). Furthermore, the expression of β-hemolysis in the absence of identifiable genetic markers suggests the presence of unidentified hemolytic mechanisms or genetic factors (Semedo et al., 2003; Dahlén et al., 2012). This discrepancy suggests that the absence of canonical WGS targets may not always accurately predict *in vitro* phenotypic behavior (Hashem, Abdelrahman & Aziz, 2021). Enzymatic virulence genes, including *gelE* and *sprE*, were absent, consistent with the lack of gelatinase activity observed in the current investigation. While adhesin and cell-wall-associated genes (*acm, scm, ecbA, sagA,* and *sgrA*) were widespread, their presence did not consistently result in strong virulence phenotypes. This discrepancy may be attributed to variability in gene expression, phase variation, transcriptional regulation by environmental signals, or the influence of mobile genetic elements. These findings align with previous reports suggesting that *E. faecium* evolution prioritizes hospital-adapted colonization and antimicrobial resistance over enzyme-mediated virulence (Arias & Murray, 2012; Brodrick et al., 2016) (Table 2 and Table S11).

**Table 2:** Biofilm associated with *empB*, *empC*, *esp*, and *hyl* genes of VREfm isolates.

**Biofilm formation**	Non biofilm	Weak	Moderate	Strong	Total
*esp* ** (%)**	0	2.2	2.2	0	4.3
**Chi-Square** ***p*** **-value**	0.942				
*hyl* ** (%)**	0	0	2.2	4.3	6.5
**Chi-Square** ***p*** **-value**	0.013				
*empB* ** (%)**	0	15.22	10.87	2.2	28.26
**Chi-Square** ***p*** **-value**	0.747				
*empC* ** (%)**	4.3	30.43	28.26	8.70	71.74
**Chi-Square** ***p*** **-value**	0.747				

**Notes.**

*p* ≤ 0.05 was considered statistically significant.

No correlation was assessed between CRISPR-Cas loci and antimicrobial resistance genes, and prophages were also variable among the VREfm studied. All isolates obtained from three hospitals in this investigation showed a significant incidence of multidrug-resistant enterococci. Whole-genome sequencing (WGS) is a technique for epidemiological research and infection control that could be used to identify transmission periods. Our data sheds light on the *E. faecium* epidemic that the three hospitals are experiencing (Pinholt et al., 2019). This work represents a significant advancement in using WGS to investigate VREfm resistance and the relationships between genotype and phenotype. WGS will aid in defining enterococci-related antimicrobial resistance.

Furthermore, while the current study provides a detailed genetic and phenotypic characterization, future large-scale investigations incorporating high-resolution genomic analysis could further clarify the evolutionary dynamics and dissemination of VREfm. Such efforts would be essential for a more comprehensive understanding of the pathogen behavior in various healthcare settings.

## Conclusions

Our findings demonstrate the high prevalence of VREfm isolates in three tertiary hospitals in Thailand. Our findings revealed that all VREfm isolates were resistant to multiple antimicrobial drugs, especially vancomycin and teicoplanin. The presence of genes associated with virulence factors and biofilm formation highlights the potential severity of VREfm infections. Interestingly, novel ST2331 was found in this study. Furthermore, control measures are crucial to prevent the spread of VREfm in hospitals and other healthcare settings. Further research is needed to elucidate the roles of the identified virulence factors, explore potential therapeutic targets for VREfm infections, and assess the need for continuous monitoring by WGS.

##  Supplemental Information

10.7717/peerj.21354/supp-1 Supplemental Information 1 Phylogenetic tree based on multi-locus sequence typing (MLST) of VREfm Multi-locus sequence typing ( MLST ), s pecimens of the type *Enterococcus faecium* were obtained from body fluid, hemoculture, pus, rectal swab, sputum tissue, and urine from Hat Yai Hospital ( HTY ) , S unpasitthipasong H ospital ( SPS ) , and S ongklanagarind H ospital ( PSU )

10.7717/peerj.21354/supp-2 Supplemental Information 2 Multi-locus sequence typing (MLST) of vancomycin-resistant *Enterococcus faecium* (VREfm) isolates from three tertiary hospitals

10.7717/peerj.21354/supp-3 Supplemental Information 3 Antibiotic susceptibility of vancomycin-resistant *Enterococcus faecium* (VREfm) isolates from three tertiary care hospitals

10.7717/peerj.21354/supp-4 Supplemental Information 4 Virulence factors of vancomycin-resistant *Enterococcus faecium* (VREfm) isolates from three tertiary care hospitals

10.7717/peerj.21354/supp-5 Supplemental Information 5 Antibiotics resistance genes of vancomycin-resistant *Enterococcus faecium* (VREfm) isolates from three tertiary care hospitals

10.7717/peerj.21354/supp-6 Supplemental Information 6 Virulence factor-related genes of vancomycin-resistant *Enterococcus faecium* (VREfm) isolates from three tertiary care hospitals

10.7717/peerj.21354/supp-7 Supplemental Information 7 Mutation genes of vancomycin-resistant *Enterococcus faecium* (VREfm) isolates from three tertiary care hospitals

10.7717/peerj.21354/supp-8 Supplemental Information 8 Prophage of vancomycin-resistant *Enterococcus faecium* (VREfm) isolates from three tertiary care hospitals

10.7717/peerj.21354/supp-9 Supplemental Information 9 CRISPR-Cas of vancomycin-resistant *Enterococcus faecium* (VR Efm ) isolates from three tertiary care hospitals

10.7717/peerj.21354/supp-10 Supplemental Information 10 Integrons type of vancomycin-resistant *Enterococcus faecium* (VREfm) isolates from three tertiary care hospitals

10.7717/peerj.21354/supp-11 Supplemental Information 11 Locus of multilocus sequence type (MLST) of vancomycin-resistant *Enterococcus faecium* (VREfm) isolates from three tertiary care hospitals

10.7717/peerj.21354/supp-12 Supplemental Information 12 Biofilm phenotypes associated with *empB, empC, esp* , and *hyl* genes of vancomycin-resistant *Enterococcus faecium* (VREfm) isolates from three tertiary care hospitals

10.7717/peerj.21354/supp-13 Supplemental Information 13 The matrix of average nucleotide identity (ANI) values of VREfm from three hospitals

## Additional Information and Declarations

### Data Availability

The following information was supplied regarding data availability: The assembled genomes of all VREfm isolates are available at Figshare: Sianglum, Wipawadee (2025). VREfm Sequence file. figshare. Dataset. https://doi.org/10.6084/m9.figshare.29857790.v1.
