# Supplementary figures and images for: Comparative genomics of hospital-associated vancomycin-resistant Enterococcus faecium from regional tertiary hospitals in Thailand
Source: PeerJ. 2026 Jun 3;14:e21354. doi: 10.7717/peerj.21354 (PMC13242186; doi:10.7717/peerj.21354)

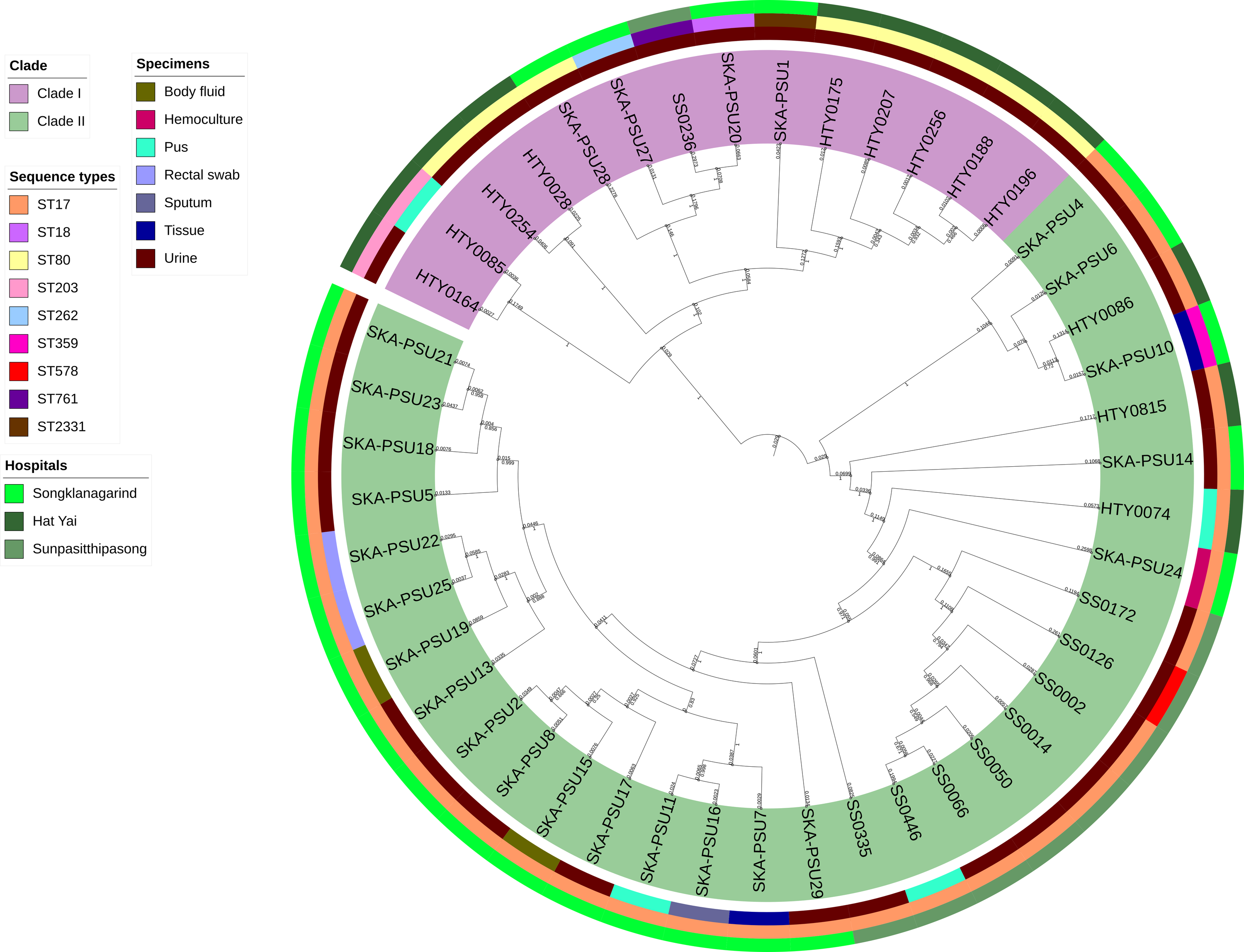

Supplement: Supplemental Information 1 — Multi-locus sequence typing ( MLST ), s pecimens of the type Enterococcus faecium were obtained from body fluid, hemoculture, pus, rectal swab, sputum tissue, and urine from Hat Yai Hospital ( HTY ) , S unpasitthipasong H ospital ( SPS ) , and S ongklanagarind H ospital ( PSU ) [file peerj-14-21354-s001.png]
